# Stereotactic Body Radiotherapy (SBRT) for Liver Tumors: A Systematic Review on the Choice of Photon Energy and Linac Flattened/Unflattened Beams

**DOI:** 10.7759/cureus.107925

**Published:** 2026-04-28

**Authors:** Kesava Ramgopal Adavikolanu, Chandramouli R, Soorej Balan Kaliyath, Thirumal M, Abhishek R K S, NagaSai Divya Kari, Pragnahitha Neerudu, Ambedkar Yadala, Madhuri Palla, Ajay Kumar Kondeti

**Affiliations:** 1 Radiation Oncology, All India Institute of Medical Sciences, Mangalagiri, Mangalagiri, IND; 2 Radiation Oncology, All India Institute of Medical Sciences, Bibinagar, Bibinagar, IND; 3 Medical Oncology, All India Institute of Medical Sciences, Mangalagiri, Mangalagiri, IND; 4 Radiation Oncology, Jawaharlal Institute of Postgraduate Medical Education & Research, Puducherry, IND; 5 Radiation Oncology, King George Hospital, Visakhapatnam, IND

**Keywords:** dosimetry, flattened photon beams, flattening filter-free (fff) beams, linac, liver sbrt, photon energy, stereotactic body radiotherapy

## Abstract

Stereotactic body radiotherapy (SBRT) enables high-precision, high-dose treatment of liver lesions with acceptable toxicity. With the introduction of flattening filter-free (FFF) beams and the use of different photon energies, there is potential for improved dosimetry and treatment efficiency; however, the optimal selection of these parameters remains uncertain. This systematic review evaluated the impact of photon energy (6 MV versus 10 MV) and beam modality (flattened versus FFF) on dosimetric quality, organ-at-risk (OAR) sparing, treatment efficiency, and clinical feasibility in liver SBRT. A comprehensive search of PubMed, Embase, and Scopus databases from January 2010 to April 2025 identified dosimetric and clinical studies comparing FFF and flattened beams as well as different photon energies. Outcomes of interest included target coverage, conformity index (CI), homogeneity index (HI), beam-on time (BOT), monitor units (MUs), and OAR doses. Risk of bias was assessed using the Newcastle-Ottawa Scale.

A total of 23 studies met the inclusion criteria with adherence to Preferred Reporting Items for Systematic Reviews and Meta-Analyses (PRISMA) guidelines. FFF beams consistently reduced beam-on time by approximately 30-60%, improved dose conformity, and enhanced OAR sparing compared with flattened beams. Among FFF modalities, 6FFF demonstrated superior conformity and steeper dose gradients, particularly for small and centrally located lesions, whereas 10FFF provided improved skin sparing and was more suitable for larger or deeper tumors. Although FFF plans required higher MUs, this was offset by significantly faster treatment delivery. Fractionation strategies were influenced by tumor size, anatomical location, and baseline liver function, with a greater number of fractions generally preferred for larger or centrally located lesions.

Overall, FFF beams, particularly 6FFF and 10FFF, offer improved treatment efficiency and favorable dosimetric outcomes in liver SBRT. The choice of beam energy and modality should be individualized based on lesion characteristics, including size, depth, and proximity to critical structures. FFF-based SBRT represents a practical and widely implementable approach, although further prospective studies are needed to support standardization of clinical practice.

## Introduction and background

Stereotactic Body Radiotherapy (SBRT) has emerged as an advanced radiotherapeutic technique for the precise and high-dose treatment of various malignancies, including primary and metastatic liver tumors [1,2]. SBRT delivers ablative radiation doses over a limited number of fractions, using steep dose gradients to spare adjacent normal tissues [3-5]. This approach has shown promise for hepatocellular carcinoma (HCC) and liver metastases, especially in patients who are not surgical candidates or for whom other local therapies such as radiofrequency ablation (RFA) or transarterial chemoembolization (TACE) are unsuitable [6-9]. 

Unlike conventional radiotherapy, SBRT requires exceptional accuracy in target localization, dose delivery, and sparing of normal liver parenchyma due to the inherent radiosensitivity and regenerative capacity of the liver. The dosimetric quality of SBRT depends on several factors, including treatment planning algorithms, motion management strategies, fractionation regimens, and critically, the choice of beam modality and energy [10,11]. 

Traditionally, photon beams used in linear accelerators include a flattening filter (FF) to produce a uniform dose distribution across the field. However, the development of flattening filter-free (FFF) technology has revolutionized modern SBRT. FFF beams allow for higher dose rates, significantly reducing beam-on time (BOT), which in turn minimizes the risk of intra-fraction motion, an especially important consideration for liver SBRT, given respiratory-induced organ motion [12]. Moreover, FFF beams offer a sharper dose falloff and reduced peripheral dose, which may enhance target conformity and OAR sparing. However, their non-uniform beam profile and potential increase in monitor units (MUs) raise concerns regarding dose homogeneity and delivery efficiency [13]. 

In parallel, the choice of photon energy typically between 6 MV and 10 MV has implications for depth dose characteristics, skin sparing, and the magnitude of lateral scatter, all of which may impact both tumor control and toxicity. Lower energy beams (e.g., 6 MV) often exhibit sharper dose gradients and are preferred in small, central-seated targets, while higher energies (e.g., 10 MV) may be beneficial in reducing surface dose but pose a higher risk of neutron contamination and increased transmission through tissues [14]. 

Recent advances in treatment planning systems (TPS) and delivery platforms, such as Volumetric Modulated Arc Therapy (VMAT), Dynamic Conformal Arc (DCA), and Intensity-Modulated Radiotherapy (IMRT), have enabled precise execution of SBRT with either FF or FFF beams and variable photon energies [15]. However, there remains a lack of consensus in the clinical community regarding the optimal combination of beam type and energy, particularly in the setting of liver SBRT. 

Several dosimetric studies and clinical reports have compared FFF vs. FF beams and 6 MV vs. 10 MV photon energy in various anatomical sites, with lung SBRT receiving the most attention [16]. Although SBRT has been successfully applied across several anatomical sites such as the lung, spine, and liver, each presents unique physical and biological challenges that demand site-specific optimization [17]. Lung SBRT focuses on managing respiratory motion and tissue heterogeneity at air-tissue interfaces, requiring robust image guidance, motion management, and dose conformity to avoid pulmonary toxicity [18]. Spine SBRT, in contrast, emphasizes steep dose gradients near the spinal cord to prevent radiation myelopathy, necessitating sub millimeter precision, rigid immobilization, and advanced image-guided verification [19]. Liver SBRT combines elements of both, requiring management of complex respiratory and cardiac motion, while ensuring sparing of radiosensitive hepatic tissue and nearby gastrointestinal organs. The liver’s limited regenerative capacity and vascular anatomy make normal-tissue protection particularly critical [20]. 

However, liver SBRT poses unique challenges due to the organ’s mobility, heterogeneous composition, and proximity to radiosensitive structures such as the stomach, bowel, and kidneys [6-9]. Thus, findings from other sites cannot be directly extrapolated to the liver. 

Despite increasing use of FFF beams, no prior systematic review has specifically evaluated both photon energy selection and beam modality in liver SBRT. Hence, this systematic review aims to critically analyze the existing literature on SBRT for liver lesions, with a focus on the impact of photon energy selection and beam modality (FF vs. FFF) on treatment quality. Specifically, we evaluate outcomes related to target coverage, dose conformity, OAR sparing, beam-on time, and overall treatment efficiency, and provide clinical insights into the selection of the most appropriate beam configuration based on the evidence to date. 

## Review

Methods

Protocol and Registration 

This systematic review was conducted in adherence with the Preferred Reporting Items for Systematic Reviews and Meta-Analyses (PRISMA) guidelines [21]. The review protocol was prospectively registered in the International Prospective Register of Systematic Reviews (PROSPERO; CRD420251060403; https://www.crd.york.ac.uk/PROSPERO/view/CRD420251060403). 

Objectives

The primary objective of this systematic review was to evaluate the dosimetric advantages of FFF photon beams compared with conventional FF photon beams in SBRT for liver lesions. Specifically, the analysis focused on key parameters including target coverage, OAR sparing, treatment delivery efficiency as measured by BOT, and the number of MUs required for treatment delivery. These parameters were selected to comprehensively assess both plan quality and treatment efficiency.

The secondary objective was to compare different photon energies, particularly 6 MV versus higher energies such as 10 MV and others, in terms of their impact on the same dosimetric and delivery-related outcomes. This comparison aimed to determine whether photon energy selection influences treatment quality, normal tissue protection, and delivery efficiency in liver SBRT.

Eligibility Criteria

Eligibility criteria were defined to include studies involving patients with liver tumors treated using photon-based SBRT, with explicit comparisons between FF and FFF beams or between different photon energies. Eligible studies were required to report relevant dosimetric or clinical outcomes, including planning target volume (PTV) coverage, conformity index (CI), homogeneity index (HI), OAR doses, BOT, and MUs. Only studies published in English between January 2010 and April 2025 were considered. Exclusion criteria included non-SBRT studies, non-English publications, conference abstracts, and review articles.

A comprehensive literature search was conducted across PubMed, Embase, and Scopus databases using combinations of keywords related to “SBRT,” “liver,” “photon energy,” and “flattening filter-free” (Appendix A). The search spanned from January 2010 to April 2025. Study selection was performed independently by two reviewers based on predefined inclusion and exclusion criteria, with disagreements resolved through consensus or consultation with a third reviewer. The selection process was documented using a PRISMA flow diagram.

Data Extraction and Analysis

Data extraction was carried out using a standardized form to ensure consistency. Extracted variables included study characteristics, treatment techniques, photon energy, beam type, dose calculation algorithms, and reported outcomes. Key dosimetric indices were either directly extracted or calculated where necessary. The CI was defined as the ratio of the volume receiving the reference dose to the target volume (VIR/TV), while the homogeneity index (HI) was calculated as the ratio of the maximum isodose within the target to the reference isodose (Imax/RI). The dose gradient index (GI) was defined as the ratio of the volume receiving the prescription isodose to the volume receiving half of the prescription isodose (DVPI/DVHPI). Additionally, D700cc was defined as the dose received by 700 cc of normal liver tissue.

The risk of bias in included studies was assessed using appropriate tools based on study design. The Cochrane Risk of Bias 2.0 tool was applied for randomized controlled trials [22], while the Newcastle-Ottawa Scale (NOS) was used for observational studies [23]. The risk of missing evidence was evaluated using the Risk Of Bias due to Missing Evidence (ROB-ME) framework [24]. Furthermore, the certainty of evidence for each outcome was assessed using the Grading of Recommendations Assessment, Development and Evaluation (GRADE) approach [25].

Given the anticipated clinical and methodological heterogeneity among the included studies, a narrative synthesis was primarily conducted. Where sufficient homogeneity existed, quantitative synthesis was performed using a random-effects model, and statistical heterogeneity was evaluated using the I² statistic.

Results

Study Selection and Characteristics 

**Figure 1:**
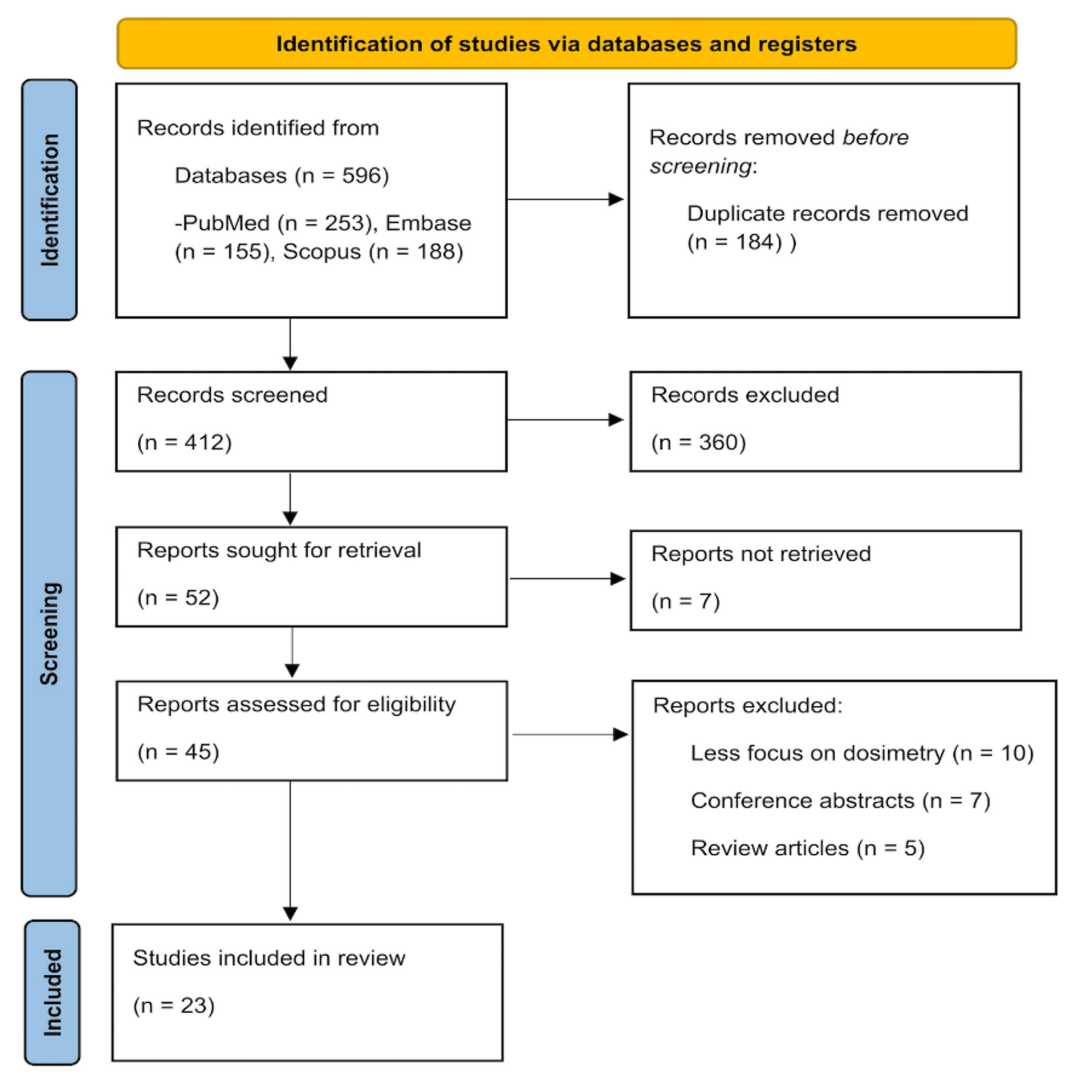
PRISMA flow diagram of the study collection PRISMA: Preferred Reporting Items for Systematic Reviews and Meta-Analyses [21].

A total of 23 studies were included after screening 412 initial records (Figure 1).

These comprised planning studies and clinical evaluations involving VMAT, IMRT, DCA, and 3 Dimensional Conformal Radiation Therapy (3DCRT) for liver SBRT. Both HCC and metastatic liver lesions were analyzed (Table 1).

**Table 1:** Primary study characteristics and results #: number of fractions or sessions over which radiotherapy will be delivered; SRS: Stereotactic Radiosurgery; PTV: Planning Target Volume; SBRT: Stereotactic Body Radiotherapy; DCA:  Dynamic Conformal Arc; DCAT: Dynamic Conformal Arc Therapy; FFF: Flattening Filter-Free; FF: Flattening Filter; CI: Conformity Index; HI: Homogeneity Index; OAR: Organ-At-Risk; BOT: Beam-On Time; MU: Monitor Units; VMAT: Volumetric Modulated Arc Therapy; IMRT: Intensity Modulated Arc Therapy; IMPT: Intensity Modulated Proton Therapy; CK: Cyber Knife; Sparc: Spot Scanning Proton Arc; AAA: Anisotropic Analytical Algorithm; MC: Monte Carlo; CC: Collapsed Cone; RTOG: Radiation Therapy Oncology Group; MLC: Multi Leaf Collimator; AXB-Acuros XB.

S. No	Study	Beam modalities	Treatment technique	Type of liver lesions	No of patients	Algorithm used for optimization	Total dose/fraction	Favorable beam modality as per the specified criteria
1	Kaur SP et al., 2025 [26]	6FFF	VMAT	-	11	Not mentioned	Not mentioned	6FFF (Better Dose Distribution and Fewer MU’s)
2	Bonù et al., 2024 [27]	6FF, 6FFF, Proton	VMAT	Both	213	Not mentioned	Range: 50–60 Gy in 3–5 fractions	Proton (spares more healthy liver) VMAT FFF (spares more healthy liver)
3	Brekner et al., 2023 [28]	6FF, 10FF, 6 FFF, 10FFF	VMAT	HCC	17	CC and MC	Not mentioned	6FFF, 10FFF (remarkable reduction of the BOT) 10FFF (Improved GTV coverage) 6FFF, 10FFF (Higher D98% of PTV) 6FFF, 10FFF (D700cc of liver is less) 10FFF (Low skin dose)
4	Suresh et al., 2022 [29]	6FF, 10FF, 6 FFF, 10FFF	3DCRT, DCA, VMAT	Both	20	AAA	40Gy/5#	VMAT FFF (Better PTV Coverage and OAR Dose) VMAT FFF (Highly Conformal Plans) VMAT FFF (Faster Treatment delivery)
5	Park et al., 2020 [30]	10 FFF	VMAT	HCC	290	Not mentioned	Range: 30–60 Gy in 3–4 fractions	SBRT 10FFF (High local control rates in patients)
6	Moon et al., 2020 [31]	6FF	DCAT, VMAT	Both	25	MC	Range: 48–60 Gy in 4–5 fractions	DCAT (shorter calculation times and delivery times with smaller MU) DCAT (should be considered preferential to VMAT in patients with poor breathing or poor coordination) VMAT (Better OAR dose) VMAT (Considered when multiple lesions)
7	Thaper et al., 2020 [32]	10FF	DCA_SSO_ VDR, VMAT	-	25	MC	Range: 25–50 Gy in 5-15 fractions	VMAT (Less OAR dose) DCA_SSO_VDR (Better CI and HI Value) DCA_SSO_VDR (Less MUs)
8	Stathakis et al., 2019 [33]	6FFF	DCAT, VMAT	-	4	MC	Range: 45–55 Gy in 3-9 fractions	DCAT (shorter calculation times and delivery times with smaller MU) DCAT (Showed comparable plan quality compared to the VMAT plans) DCAT (dosimetrically equivalent and an efficient alternative to VMAT plans)
9	Munirathinam et al., 2018 [34]	6FF, 6FFF	VMAT	Mets	8	Not mentioned	50Gy/10#	6FFF (BOT Reduction) 6FFF (Better dose distribution)
10	Ogata et al., 2017 [35]	10FFF, 10FF	3DCRT	Both	10	AAA	48Gy/4#	FFF and FFF (Similar target coverage and sparing of normal tissue) FFF (Better HI value)
11	Vieillevigne et al., 2016 [36]	6FF, 6FFF, 10FF, 10FFF	DCA, VMAT	-	Phantom	AAA	60Gy/3#	VMAT FFF (Better sparing of normal tissue) VMAT FFF (BOT Reduction)
12	Kim GJ et al., 2016 [37]	10FF, 10FFF	VMAT	-	2	Not mentioned	45Gy/3#	10 FFF (BOT Reduction)
13	Jin et al., 2016 [38]	6FFF	IMRT, VMAT	HCC	9	Not mentioned	50 or 32.5/5#	Cyberknife (Lower Mean dose for critical structures and better CI) Varian Linac (Less treatment delivery time)
14	Kim JY et al., 2015 [39]	Proton, Photon	PBT, H- IMRT, VMAT	HCC	30	Not mentioned	60Gy/10#	Proton (spares more healthy liver)
15	Stieb et al., 2015 [40]	6FFF or 10FFF	VMAT	Both	10	Not mentioned	Range: 35–60 Gy in 4–10 fractions	FFF (No unexpected toxicity occurred) FFF (time efficient and appears to be safe)
16	Esposito et al., 2015 [41]	Multiple techniques, Beam energies	3DCRT, IMRT, VMAT	Mets	5	Not mentioned	75Gy/3#	VMAT FFF (BOT Reduction)
17	Gandhi et al., 2015 [42]	Photon, Proton	VMAT, PBT	HCC	10	Not mentioned	50Gy/5#	Proton (Better Normal Liver Sparing) Proton (Less radiation toxicity)
18	Mancosu et al., 2012 [43]	FF, FFF	VMAT	Both	55	AAA (Analytical Anisotropic Algorithm)	75Gy/3#	FFF (BOT Reduction)
19	Prendergast et al., 2012 [44]	FF, FFF	VMAT, IMRT	Both	99	Not mentioned	Range: 6–20 Gy in 3–5 fractions	FFF (Improved Treatment Delivery Time) FFF (BOT Reduction)
20	Reggiori et al., 2012 [45]	10FF, 10FFF	VMAT	Mets	10	Not mentioned	75Gy/3#	10 FFF (BOT Reduction)
21	Lang et al., 2012 [46]	6FFF, 10FFF	VMAT	Both	Total 26 and 4 for liver	AAA	Range: 40–50 Gy in 4–12 fractions	VMAT FFF (BOT Reduction) VMAT FFF (Minimal patient and target shift during treatment)
22	Scorsetti et al., 2011 [47]	6FFF or 10FFF	VMAT	Mets	10	AAA	75Gy/3#	FFF (BOT Reduction) FFF (Promising tumor local control)
23	Petersen et al., 2011 [48]	Photon, Proton	IMPT, IMRT	Mets	10	AAA, PB	Range: 37.5– 50.25 Gy in 3 fractions	IMPT (Better treatment plans and dose coverage less OAR dose)

In view of heterogeneity of studies, a narrative synthesis was undertaken. The included studies demonstrated an overall moderate risk of bias, primarily driven by non-randomized study designs, small sample sizes in several analyses, and substantial confounding due to technological and patient-related heterogeneity (Appendix B).The summary table for key outcomes such as BOT reduction, CI/HI values is given as Appendix C.

Beam Modalities: FF vs. FFF Beams 

FFF beams consistently reduced BOT (by 30-60%) and improved conformity and OAR sparing compared to FF beams. Improvements in GTV coverage, liver sparing, and reduced skin dose were commonly reported. FFF beams also enabled better utilization of high dose per fraction regimens. FFF delivery allowed for faster treatment sessions, which is clinically advantageous for minimizing intra-fraction motion and improving patient throughput. 

FFF beams provided equal or superior target coverage in terms of D95%, D98%, and PTV coverage. Liver dose constraints (e.g., D700cc, mean liver dose) were better achieved using FFF beams in many studies. FFF delivery reduced the dose to healthy liver tissue, bowel, stomach, and skin in most cases and improved spatial and temporal accuracy, reducing patient movement and positional drift during long treatments. 

Photon Energy Comparison (6 MV vs. 10 MV)

6 MV FFF: Several studies favored 6FFF for its sharper penumbra, better conformity, and enhanced sparing of critical structures, particularly in small-to-medium-sized lesions, and found that 6FFF reduced D700cc of normal liver and provided improved dose gradients around PTV. 

10 MV FFF: 10FFF was advantageous in specific contexts, particularly for reducing skin dose and for larger or deep-seated lesions, where improved beam penetration was needed. 10FFF provided superior GTV coverage and reduced skin dose, especially when used with VMAT. 

Mixed beam use: A few studies, utilized both 6FFF and 10FFF in the same cohort. Their findings suggested that the choice of energy should be tailored based on PTV size, depth, and proximity to sensitive structures. Larger lesions may benefit from the deeper penetration of 10FFF, while smaller or superficial lesions may be best treated with 6FFF. 

Fractionation Schedules and Interaction With Beam Type 

Among the included studies, fractionation schedules for liver SBRT ranged from single-fraction (25-30 Gy) to multi-fraction regimens of three to five fractions (total 30-60 Gy). Most dosimetric comparisons involving FFF and FF beams used three- to five-fraction protocols, consistent with institutional liver SBRT practice. Some of the studies reported that the advantage of FFF beams, particularly reducing BOT by up to 60%, was most evident in single- or few-fraction schedules, where intrafraction motion is a greater concern. In contrast, for five-fraction regimens, FFF and FF beams produced similar CIs and HIs, though treatment efficiency still improved. These findings indicate that the dosimetric benefit of FFF delivery is amplified with higher per-fraction doses, aligning with the motion-sensitive nature of liver SBRT. 

Discussion

This systematic review comprehensively evaluated the impact of photon beam characteristics specifically FFF versus flattened FF beams and varying photon energies on the quality and efficiency of SBRT for liver lesions. Our findings indicate a consistent dosimetric advantage of using FFF beams, particularly 6FFF and 10FFF, across a broad range of clinical and planning contexts. 

Clinical Relevance of FFF Beams in Liver SBRT 

The FFF beams affirms that BOT reductions and shorter treatment durations decrease the likelihood of intra-fraction motion, which in turn improves geometric accuracy and target localization. Moreover, the potential of FFF beams to improve plan conformity and steeper dose gradients is well-supported. A more conformal dose distribution is especially important in the liver due to the organ’s proximity to radiosensitive structures like the stomach, bowel, kidneys, and spinal cord. Many studies included in our review, particularly those using VMAT-based FFF plans, reported improved CIs and lower doses to OARs, thereby enhancing the therapeutic ratio [26,27,35,49]. 

Photon Energy Considerations: 6FFF vs. 10FFF 

Across included studies, lesion size and depth definitions were heterogeneous. However, planning data generally classified small and superficial lesions as those with PTV <100 cm³ or located within 5 cm from the liver surface, and large or deep lesions as those with PTV >100-150 cm³ or situated >5 cm below the capsule. These thresholds, although not universal, provide practical guidance for energy selection in clinical planning. 

A key objective of this review was to investigate whether the choice of photon energy (typically 6 MV or 10 MV) influences dosimetric quality in liver SBRT. The results suggest that 6FFF beams are generally preferable for small, centrally located, or shallow lesions. This is primarily due to their sharper penumbra and enhanced dose conformity, which facilitate more precise dose sculpting and improved sparing of healthy liver tissue [33,46,48]. 

10FFF beams offer benefits in select situations, particularly when deeper tissue penetration is needed, such as in large or deeply located lesions. They also demonstrate improved skin sparing due to the beam's higher energy. However, these benefits are sometimes offset by a broader penumbra and reduced conformity compared to 6FFF. Hence, the choice between 6FFF and 10FFF should be individualized based on tumor characteristics, anatomical location, and institutional planning capabilities [42,50]. 

Dosimetric Trade-offs and Planning Complexity

While FFF beams were occasionally associated with slightly higher MUs, this increase was not clinically concerning and was often offset by the significantly reduced BOT. Although FFF beams offer clear advantages in terms of reduced BOT, improved treatment efficiency, and sharper dose gradients, certain trade-offs should be acknowledged. Several studies reported a modest increase in MUs with FFF delivery, particularly for large or complex target geometries. This increase, however, did not translate into clinically significant differences in treatment time or patient dose due to the higher dose rate of FFF beams [37,43]. Additionally, dose homogeneity may be slightly reduced in large or asymmetric fields because of the non-uniform intensity profile inherent to FFF beams [13]. Nevertheless, modern planning algorithms (Analytical Anisotropic Algorithm (AAA), Monte Carlo) and VMAT optimization effectively mitigate these effects, maintaining acceptable HIs and CIs [51]. Therefore, while FFF beams are not universally superior in all scenarios, their clinical efficiency and motion-management advantages outweigh these minor limitations in most liver SBRT contexts. Importantly, treatment techniques such as VMAT and IMRT can exploit the high dose rates of FFF beams without compromising dosimetric quality. Additionally, several studies reported that FFF beams offered reduced peripheral dose and improved sparing of non-target liver volume (e.g., D700cc), a parameter increasingly recognized for predicting hepatic toxicity in SBRT. This makes FFF particularly valuable in patients with pre-existing liver dysfunction or cirrhosis common comorbidities in hepatocellular carcinoma [31,43,45,52]. 

Fractionation Schedules

Across the included studies, fractionation schedules ranged from single-fraction high-dose treatments (e.g., 25-30 Gy × 1) to more conventional regimens of three to five fractions totalling 30-60 Gy. The benefit of FFF beams was most pronounced in single- and few-fraction regimens, where reduced BOT directly minimized intra-fraction motion and improved treatment efficiency. For multi-fraction schedules (three to five fractions), FFF delivery continued to enhance workflow and patient comfort but contributed less to dosimetric differences compared with flattened beams. Some of the included studies specifically noted greater BOT reduction and improved conformity with 6FFF in hypo fractionated regimens [28,43,53]. These findings suggest that the clinical advantage of FFF delivery is fractionation-dependent, being most impactful when per-fraction doses and intrafraction motion risks are highest. 

Heterogeneity and Limitations 

There was notable heterogeneity across the included studies in terms of planning systems and optimization algorithms, fractionation regimens (ranging from one to five fractions), target sizes and anatomical locations, and respiratory motion management strategies. Although a meta-analysis was not feasible due to this variability, the consistent trends observed across multiple studies strengthen the validity of our conclusions. 

A key limitation of the current review is that most included studies focused exclusively on dosimetric parameters, with limited or absent reporting of clinical outcomes such as local control, hepatic toxicity, or overall survival. Consequently, while the dosimetric advantages of FFF beams are evident, their translation into tangible clinical benefit remains to be validated. Future prospective trials should integrate both dosimetric and clinical endpoints to determine whether improved conformity, lower organ doses, and shorter BOTs correlate with enhanced tumor control and reduced hepatic or gastrointestinal toxicity in real-world settings. Publication bias could not be formally assessed due to the heterogeneity and predominance of dosimetric studies; however, the possibility of selective reporting of favorable outcomes cannot be excluded. Comparison across different fractionation regimens introduces radiobiological variability, which may influence OAR tolerance and limits direct dosimetric comparability.

Future Directions

Prospective studies with clinical endpoints, cost-effectiveness analyses, and individualized beam selection strategies are warranted to optimize liver SBRT outcomes. 

## Conclusions

This review shows that FFF photon beams, particularly 6FFF and 10FFF, offer notable dosimetric and treatment efficiency benefits for liver SBRT. They reduce treatment time, improve conformity, and enhance OAR sparing. 6FFF is advantageous for small, central lesions, while 10FFF suits larger or deep-seated tumors. The choice of beam energy should be patient- and lesion-specific. In summary, the findings of this review support the integration of FFF beam technology, particularly 6FFF and 10FFF, into routine SBRT planning for liver tumors. The selection of beam energy and configuration should be individualized, guided by tumor size, depth, anatomical proximity to critical organs, and available technology. Future prospective trials reporting both dosimetric and clinical outcomes are essential to validate these observations and develop consensus-based clinical guidelines. 

## Figures and Tables

**Figure 2:**
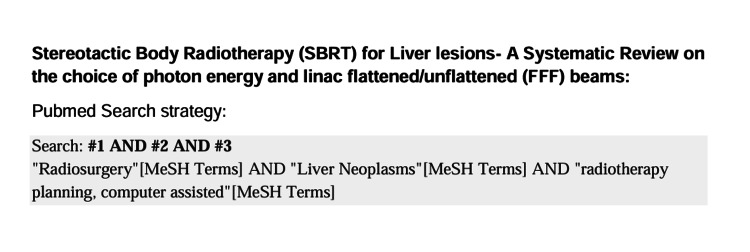
Search strategy with MeSH terms

## Appendices

Appendix A 

Appendix B 

**Table 2:** Risk of bias assessment for the included studies

S. No	Study	Design	Sample size	Selection bias	Performance bias	Detection bias	Confounding	Key limitations	Overall RoB
1	Kaur et al., 2025 [26]	Retrospective planning	33	Moderate	Low	Low	Moderate	Mixed sites, retrospective design	Moderate
2	Bonù et al., 2024 [27]	Multicenter retrospective	213 lesions	Moderate	Moderate	Low	High	Technique selection bias	Moderate
3	Brekner et al., 2023 [28]	Retrospective planning	44	Moderate	Low	Low	Moderate	Lesion heterogeneity	Moderate
4	Suresh et al., 2022 [29]	Planning study	20	Moderate	Low	Low	Moderate	Multiple techniques compared	Moderate
5	Park et al., 2020 [30]	Retrospective clinical	290	Moderate	Moderate	Low	High	Clinical heterogeneity	Moderate–High
6	Moon et al., 2020 [31]	Retrospective planning	25	Moderate	Low	Low	Moderate	Planner variability	Moderate
7	Thaper et al., 2020 [32]	Retrospective planning	25	Moderate	Moderate	Low	Moderate	Technique heterogeneity	Moderate
8	Stathakis et al., 2019 [33]	Planning study	4	Moderate	Low	Low	Low–Moderate	Very small sample	Moderate
9	Munirathinam et al., 2018 [34]	Planning study	8	High	Low	Low	Moderate	Very small sample	Moderate–High
10	Ogata et al., 2017 [35]	Planning study	10	Moderate	Low	Low	Low–Moderate	Small sample	Moderate
11	Vieillevigne et al., 2016 [36]	Planning (phantom + clinical)	24 PTVs	Moderate	Low	Low	Moderate	Mixed datasets	Moderate
12	Kim et al., 2016 [37]	Planning study	5	High	Low	Low	Moderate	Extremely small sample	High
13	Jin et al., 2016 [38]	Planning study	9	Moderate	Moderate	Low	High	Platform comparison bias	Moderate–High
14	Kim et al., 2015 [39]	Planning study	30	Moderate	Low	Low	High	Proton vs photon bias	Moderate
15	Stieb et al., 2015 [40]	Retrospective clinical	84	Moderate	Moderate	Low	High	Mixed tumor sites, short FU	Moderate
16	Esposito et al., 2015 [41]	Multicenter planning	5	High	High	Low	High	Inter-institution variability	High
17	Gandhi et al., 2015 [42]	Model + validation	10	Moderate	Moderate	Low	High	Modeling assumptions	Moderate–High
18	Mancosu et al., 2012 [43]	Prospective cohort	55	Low–Moderate	Low	Low	Moderate	No comparator arm	Moderate
19	Prendergast et al., 2012 [44]	Retrospective cohort	99	Moderate	Moderate	Low	High	Temporal bias	Moderate–High
20	Reggiori et al., 2012 [45]	Planning study	10	Moderate	Low	Low	Moderate	Small cohort	Moderate
21	Lang et al., 2012 [46]	Clinical + planning	26	Moderate	Low	Low	Moderate	Mixed tumor types	Moderate
22	Scorsetti et al., 2011 [47]	Prospective cohort	10	Low–Moderate	Low	Low	Moderate	No comparator arm	Moderate
23	Petersen et al., 2011 [48]	Planning study	10	High	Low	Low	High	Proton vs photon bias	High

Appendix C 

**Table 3:** Summary table of BOT reduction, CI, and HI BOT: beam-on time; CI: conformity index; HI: homogeneity index; FF: flattening filter; FFF: flattening filter free.

Study	BOT Reduction / BOT Data	Conformity Index (CI)	Homogeneity Index (HI)
Kaur et al., 2025 [26]	Not reported	Not reported	Not reported
Bonù et al., 2024 [27]	Not reported	0.74–0.8	Not reported
Brekner et al., 2023 [28]	78.4% BOT reduction (FFF)	1.18	Minor improvement
Suresh et al., 2022 [29]	≈ 75% BOT reduction (FFF)	1.19	0.08 ± 0.03
Park et al., 2020 [30]	No BOT/CI/HI data	—	—
Moon et al., 2020 [31]	~20% reduction (DCAT faster)	VMAT 1.03, DCAT 1.05	VMAT 1.07, DCAT 1.08
Thaper et al., 2020 [32]	≈ 70% reduction (DCA SSO VDR faster)	VMAT ≈ 1.05, DCA SSO VDR ≈ 1.10	—
Stathakis et al., 2019 [33]	≈ 59–60% reduction (DCAT faster)	VMAT ≈ 1.33, DCAT ≈ 1.42	—
Munirathinam et al., 2018 [34]	~30–40% reduction (FFF faster)	FFF 0.91, FB 0.79	≈ 1.06
Ogata et al., 2017 [35]	70–75% reduction (FFF faster)	FFF ≈ 1.05, FF ≈ 1.02	—
Vieillevigne et al., 2016 [36]	55–75% reduction (FFF faster)	≈ 1.0–1.2	Nearly identical
Kim et al., 2016 [37]	50–70% reduction (FFF faster)	FFF CI 0.95, FF CI 0.94	FFF HI 1.12, FF HI 1.11
Jin et al., 2016 [38]	50% reduction (VMAT FFF)	CK CI 1.07, Varian Linac 1.19	Nearly identical
Kim et al., 2015 [39]	Not quantified	≈ 1.45	≈ 1.03
Stieb et al., 2015 [40]	40–50% reduction (FFF faster)	FFF CI 1.15, FF CI 1.13	FFF HI 1.38, FF HI 1.3
Esposito et al., 2015 [41]	2.4–57 min → up to 90% reduction (VMAT FFF)	1.10–1.25	1.07–1.53 (mean ≈ 1.16 ± 0.27)
Gandhi et al., 2015 [42]	Not reported	—	—
Mancosu et al., 2012 [43]	45% reduction (FFF faster)	FFF CI 1.14, FF CI 1.15	FFF HI 1.1, FF HI 1.09
Prendergast et al., 2012 [44]	Not reported	—	—
Reggiori et al., 2012 [45]	70% reduction (FFF faster)	FFF CI 1.19, FF CI 1.04	Nearly identical
Lang et al., 2012 [46]	≈ 70% reduction (FFF faster)	1.10	Not reported
Scorsetti et al., 2011 [47]	≈ 70% reduction (FFF faster)	1.20	Not reported
Petersen et al., 2011 [48]	Not reported	—	—
